# Computational Design of a Carbon Nanotube Fluorofullerene Biosensor

**DOI:** 10.3390/s121013720

**Published:** 2012-10-12

**Authors:** Tamsyn A. Hilder, Ron J. Pace, Shin-Ho Chung

**Affiliations:** 1 Computational Biophysics Group, Research School of Biology, Australian National University, Acton, ACT 0200, Australia; E-Mail: shin-ho.chung@anu.edu.au; 2 Biophysical Chemistry, Research School of Chemistry, Australian National University, Acton, ACT 0200, Australia; E-Mail: ron.pace@anu.edu.au

**Keywords:** carbon nanotube, biosensor, fluorofullerene, molecular dynamics, distributional molecular dynamics, proof-of-concept

## Abstract

Carbon nanotubes offer exciting opportunities for devising highly-sensitive detectors of specific molecules in biology and the environment. Detection limits as low as 10^−11^ M have already been achieved using nanotube-based sensors. We propose the design of a biosensor comprised of functionalized carbon nanotube pores embedded in a silicon-nitride or other membrane, fluorofullerene-Fragment antigen-binding (Fab fragment) conjugates, and polymer beads with complementary Fab fragments. We show by using molecular and stochastic dynamics that conduction through the (9, 9) exohydrogenated carbon nanotubes is 20 times larger than through the Ion Channel Switch ICS™ biosensor, and fluorofullerenes block the nanotube entrance with a dissociation constant as low as 37 pM. Under normal operating conditions and in the absence of analyte, fluorofullerenes block the nanotube pores and the polymer beads float around in the reservoir. When analyte is injected into the reservoir the Fab fragments attached to the fluorofullerene and polymer bead crosslink to the analyte. The drag of the much larger polymer bead then acts to pull the fluorofullerene from the nanotube entrance, thereby allowing the flow of monovalent cations across the membrane. Assuming a tight seal is formed between the two reservoirs, such a biosensor would be able to detect one channel opening and thus one molecule of analyte making it a highly sensitive detection design.

## 1. Introduction

Nanotubes have already attracted attention for use as highly sensitive biosensors due to their unique electrical and mechanical properties. Functionalized carbon nanotubes that display distinct characteristics triggered by changes in their surrounding environment offer exciting opportunities for the detection of specific molecules, particularly in biological and environmental samples [1]. Already, nanotube-based biosensors have shown detection limits as low as 10^−11^ M (for reviews see references [2] and [3]). The majority of nanotube-based sensors exploit their electrical properties, but attention has been drawn to sensors which make use of the nanotube pore for ionic conduction [4–9]. Most reported carbon nanotube-based biosensors are enzyme-modified electrodes for detecting different biomolecules (such as glucose) and environmental pollutants (such as organo-phosphate pesticides) [3]. Owing to their sensitivity, these biosensors may lead to numerous technological advances in the medical industry.

The excellent electrical property of carbon nanotubes has led to their incorporation into numerous biosensor designs. Moreover, since the current flows along the nanotube surface, the electrical conductance of the nanotube is highly sensitive to chemical changes on its sidewalls [10]. In these devices the binding of a target molecule to the carbon nanotube surface is detected by a change in electrical signal, such as through a change in conductance [11]. These types of biosensors have been used to detect *E. coli* [11], or glucose molecules [12,13]. Moreover, they can also be used to study dynamic processes, such as the release of ATP from living cells [10]. Sotiropoulou and Chaniotakis [12] used an array of multi-walled carbon nanotubes grown on a platinum substrate. When glucose oxidase enzymes are immobilized to the nanotube ends a change in electrical current occurs. By measuring currents that flow across the nanowire, they demonstrate the detection of glucose to 0.19 mM, which is among the best reported values for glucose biosensors. Carbon nanotube forests have also been developed to detect various proteins, such as human serum albumin [14], prostate specific antigen [15] and horseradish peroxidase [16]. Antibodies are covalently attached to the carboxylated ends of the carbon nanotubes and the specific protein is detected by a change in electrical current. For example, these forests could detect 1 nM of human serum albumin [14]. Liu *et al.* [17] demonstrated the potential of single-wall carbon nanotubes to detect volatile organic compounds in human breath to diagnose lung cancer. Their results show that the resistance of the nanotubes increases when exposed to both polar and non-polar molecules. Similarly, resistance changes of carbon nanotubes have also been used to detect viruses [18] and the toxic gases NO_2_ and NH_3_ [19]. Recently, Park *et al.* [20] developed a biosensor which mimics the canine nose comprised of olfactory nanovesicle fused carbon nanotube transistors which can detect hexanal, an indicator of oxidation of food, at 1 fM concentration by monitoring conductance change of the nanotube. As well as their unique electrical properties, nanotubes are also widely investigated due to their remarkable mechanical properties. In such sensors, the nanotube acts as a cantilever which is sensitive to the molecule of interest [21]. Similar to the change in electrical signal generated by the immobilization of specific proteins onto the nanotube, mechanical deformations of the nanotube cantilever can also induce changes to electrical conductance [21].

One of the simplest uses of nanotubes for sensing applications is the detection of analytes due to a change in transmembrane current, such as proposed in this paper. Carbon nanotube membranes with nanotube diameters of approximately 7.5 nm in diameter have been used to detect streptavidin [4] and desthiobiotin [5] when the ionic flux is reduced by a factor of 15 and 24, respectively. Nednoor *et al.* [6] also modulate ionic flux through their array of aligned carbon nanotubes 7 nm in diameter embedded in a polystyrene matrix by phosphorylation. Phosphorylation and dephosphorylation of synthetic peptides at the nanotube entrance modulate ionic flux as a result of antibody attachment by reversing the charges at the nanotube entrance. Gold nanotubes have also been used to detect specific proteins [7–9]. Siwy *et al.* [9] have embedded conically shaped gold nanotubes with diameters 5–9 nm in a polymeric membrane. Proteins are detected when they occlude the nanotube entrance, thus preventing the flow of ionic currents. Similarly, Kobayashi and Martin [7] fabricated membranes constructed from gold nanotubes with inside diameters ranging from 1 to 4 nm and achieved detection limits of 10^−11^ M by measuring the change in transmembrane current when an analyte effectively plugs the nanotube entrance. A disadvantage with these size-based selectivity designs is that the analyte species must have molecular dimensions comparable to the inside diameter of the nanotubes; thus, in practice, it would be difficult to distinguish between molecules of a similar size. Moreover, only proteins with comparable diameters to the nanotube mouth can be detected. In contrast, Steinle *et al.* [8] mimicked ligand-gated ion channels where their gold nanotube embedded membrane is switched from the “off” state to the “on” state in response to a chemical stimulus. The “off” state is obtained by making the membranes hydrophobic so that water and ions cannot enter the pore. Then, by adding sufficiently high concentrations of hydrophobic ionic species, such as a drug, the membrane switches to the “on” state since the pores fill with water and electrolyte.

The Ion Channel Switch ICS™ biosensor [22] makes use of ionic flow through the bacterial channel gramicidin-A. Gramicidin-A is a polypeptide consisting of 15 amino acid residues which forms a narrow pore of approximately 2 Å in radius. It was one of the first antibiotics used clinically as it forms a channel across bacteria cell membranes and selectively conducts monovalent cations [23,24]. This biosensor has been used for the detection of low molecular weight drugs, proteins and microorganisms [25]. It is constructed from a gold electrode to which is tethered a lipid membrane containing gramicidin ion channels linked to antibodies. The switch has a high gain, where a single channel conducts up to a million ions per second, and can detect analytes from sub-picomolar to micromolar concentrations in less than 10 minutes [22]. There are 10^8^ gramicidin channels per cm^2^, so that each electrode area of 0.03 cm^2^ contains 3 × 10^6^ channels with approximately half of these being dimerized [26] or open so that the measured current is of the order of microamperes. These currently used biosensors respond to concentrations as low as 10 fM [26]. Work is continuing to miniaturize these ICS™ biosensors, but electrode sizes of 1 μm radius only contain a few ion channels and therefore measured current is of the order of picoamperes and measurement noise becomes a significant issue [26].

Here we illustrate a design, based on a similar principle to the ICS™ biosensor [22], of an ultra-sensitive detector, comprised of an array of functionalized carbon nanotubes and fluorinated fullerenes (fluorofullerenes). Under normal conditions all carbon nanotubes are blocked by the fluorofullerenes. The fluorofullerenes are attached to Fragment antigen-binding (Fab fragments) and therefore not only block the nanotube pore but also act as recognition elements. Upon detection of some analyte the fluorofullerenes are stripped from the nanotube entrance, allowing monovalent cations to conduct at a rate of 82 pA per nanotube. We demonstrate, using molecular and stochastic dynamics, the permeation characteristics of monovalent cations through the unblocked carbon nanotubes, construct the free energy of binding of two fluorofullerenes to the nanotube pore and calculate their respective binding affinity. Finally, we outline the construction and function of our proof-of-concept biosensor design.

## 2. Theoretical Section

Using molecular dynamics (MD) and distributional molecular dynamics (DMD) simulations we examine a (9, 9) single-walled carbon nanotube (CNT) with an effective radius of 4.53 Å and length of 36 Å embedded in a lipid bilayer separating two reservoirs. The nanotube is terminated with hydrogen atoms with a partial charge of 0.115e, and −0.115e on carbon atoms directly bonded to these hydrogen atoms [27]. In addition, two regions on the nanotube outside surface are hydrated (exohydrogenated). These two regions are located from z = −11.8 to −6.9 Å and from z = 6.9 to 11.8 Å. Further details of the methodology can be found in our earlier papers [28–30].

### 2.1. Molecular Dynamics

MD simulations are used to calculate the profile of potential of mean force (PMF) of (i) a potassium or chloride ion traversing through the CNT and (ii) two fluorofullerenes (C_60_F_36_ and C_60_F_60_) binding to the (9, 9) CNT. All MD simulations are performed using NAMD 2.8 and visualized using VMD 1.9 [31,32]. All simulations use the CHARMM27 force field [33,34] and TIP3P water model, a constant pressure (1 atm) and temperature 310 (K). Atom coordinates and topology file for the C_60_ fullerene were taken from the NAMD mailing list [35]. Parameters for the fluorine atoms were obtained from CHARMM36 force field [33,34,36] and Dunlap *et al.* [37]. The carbon and fluorine atoms of the fluorofullerenes are given a partial charge of 0.071e and −0.071e, respectively [38]. The nanotube is embedded in a 3-palmitoyl-2-oleoyl-D-glycero-1-phosphatidylethanolamine (POPE) lipid bilayer separating two reservoirs. The PMF for the potassium and chloride ions is conducted using umbrella sampling at an ionic concentration of 0 mM to avoid double-counting of the ion-ion interactions in subsequent DMD simulations in which the ion-ion interactions are explicitly dealt with using macroscopic electrostatics [28]. The effect of this is discussed in our earlier paper [30]. Therefore, the simulation contained either one potassium or one chloride ion.

The PMF for the binding of the fluorofullerene to the carbon nanotube is obtained using umbrella sampling. The fluorofullerenes were initially placed at the nanotube entrance and equilibrated for 1 ns. Using the equilibrated nanotube-fullerene structure we generate sampling windows by performing steered molecular dynamics. A force constant of 10 kcal/mol/Å^2^ is applied to pull the fluorofullerene away from the nanotube entrance. The channel central axis is used as the reaction coordinate. The pulling generates a continuous number of configurations along the permeation pathway so that we can construct an umbrella sampling window every 0.5 Å. During umbrella sampling the centre of mass of the fluorofullerene is confined to be within a cylinder of 8 Å centred on the channel axis and beyond this a harmonic constraint of 20 kcal/mol/Å^2^ is applied. In addition, a force constant of 10 kcal/mol/Å^2^ is applied in the *z* direction to constrain the centre of mass to the sampling window. The centre of mass coordinates of the fluorofullerene are saved every 0.5 ps. The weighted histogram analysis method [39,40] and centre of mass coordinates are used to construct the PMF along the *z* direction. Each sampling window is run for 5 ns. The PMF is shown to converge as the depth changes by less than 0.5 kT when simulations are run for a further 1 ns. The dissociation constant (*K_d_*) in the unit of molar is estimated to be [41,42]:

(1) $$K_{d}^{-1}=1000N_{A}\pi R^{2}\int_{z_{1}}^{z_{2}}\exp(-W(z)/k_{B}T)dz$$

where *W*(*z*) is the 1D PMF with the zero point located at bulk, 1,000 *N_A_* is used to convert from m^3^ to L/mol, *k_B_* and *T* are Boltzmann's constant and temperature, respectively, and *z*_1_ is in the binding pocket and *z*_2_ is in the bulk where the PMF vanishes. The centre of mass of the nanotube channel is set at 0 Å.

### 2.2. Distributional Molecular Dynamics

DMD simulations [28–30] are used to determine the current-voltage profile of potassium ions traversing through the carbon nanotube. DMD allows us to reproduce the distribution of ion trajectories from MD as closely as possible but enables simulations that are two to three orders of magnitude faster than classical MD simulations.

We first determine the free energy profile and frictional and random force (friction kernel) at discrete segments within the channel using MD. Then, we incorporate these results into stochastic dynamics simulations based on the non-linear generalized Langevin equation to determine the current-voltage profile, and ion binding sites within the channel. The simulation space is separated into two regions. In the first region, the pore (nanotube) is divided into thin slices and the forces acting on each ion and diffusion coefficient are obtained from the free energy and friction kernel generated using MD so that certain properties of the ion trajectory are reproduced in DMD. Macroscopic electrostatics is used for ion-ion interactions within the pore. We use a dielectric constant of 2 for the membrane region and 60 for water within the nanotube. These values have been shown to provide excellent agreement with experimental data for biological ion channels which have pores of a similar size [43]. In the second region, the reservoir, normal Brownian dynamics and macroscopic electrostatics is performed [44].

## 3. Results and Discussion

### 3.1. Ion Conduction

**Figure 1.**
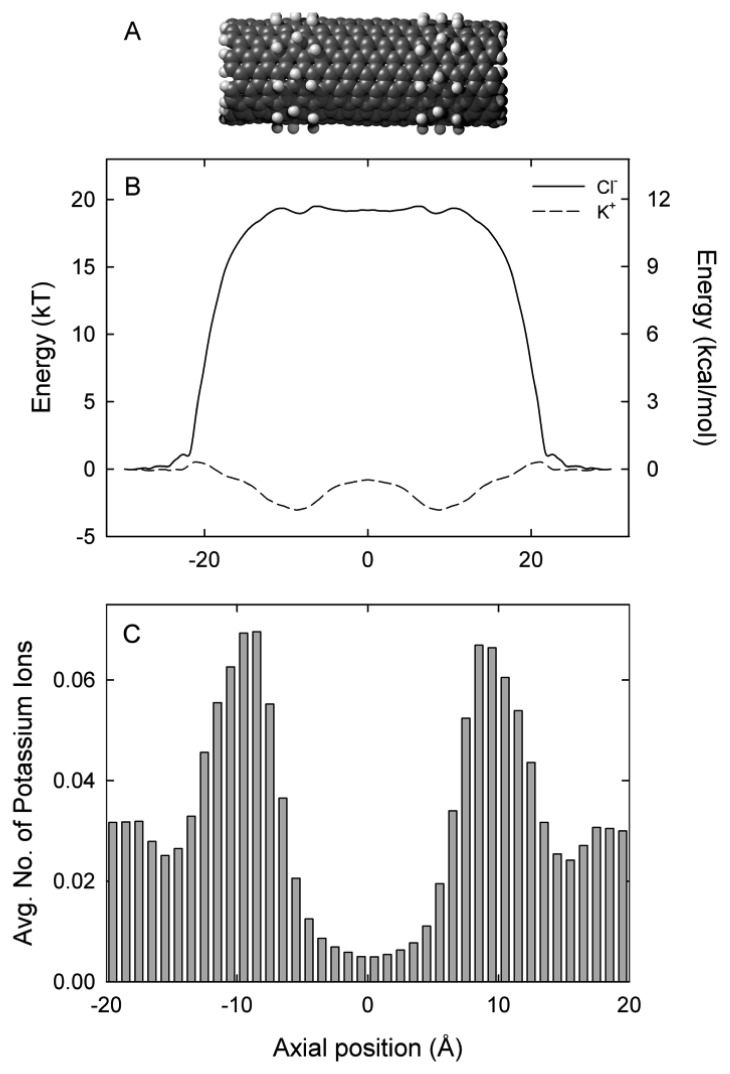
(**A**) Schematic of the (9, 9) exohydrogenated carbon nanotube with hydrogen atoms shown in light grey; (**B**) Free energy profile along the axial coordinate for potassium (K^+^) and chloride (Cl^−^) ions; (**C**) Dwell histogram of potassium ions in the absence of an applied electric potential.

The (9, 9) exohydrogenated CNT 36 Å in length is shown in Figure 1(A). Figure 1(B) shows the axial free energy encountered by potassium (K^+^) and chloride (Cl^−^) ions as they traverse the CNT. The free energy profile is derived from the three-dimensional PMF and is therefore the energy each ion encounters as it moves along the axis. Potassium ions encounter two energy wells of approximately 3 kT at the each end of the nanotube, located at ±9 Å. These wells are separated by a shallow energy barrier of 2.2 kT. In contrast, chloride ions encounter an insurmountable energy barrier of approximately 20 kT. The location of the energy wells for potassium ions align with the location of the exohydrogenated regions of the nanotube shown in Figure 1(A). In the absence of an applied potential, potassium ions tend to dwell in the centre of the two exohydrogenated regions of the nanotube (at ±9 Å), as shown in Figure 1(C). On average, 1.2 potassium ions are present in the 36 Å length nanotube. The nanotube length examined in this paper is much shorter than the smallest nanotubes produced experimentally (200 Å) [45]. To maintain low barriers for potassium ions it will be necessary to exohydrogenate repeating sections of the entire length of the long nanotube to generate an undulating profile. In the exohydrogenated sections of the nanotube an ion experiences a negatively lined pore as a result of the negative charges on the carbon atoms (−0.115e).

**Figure 2.**
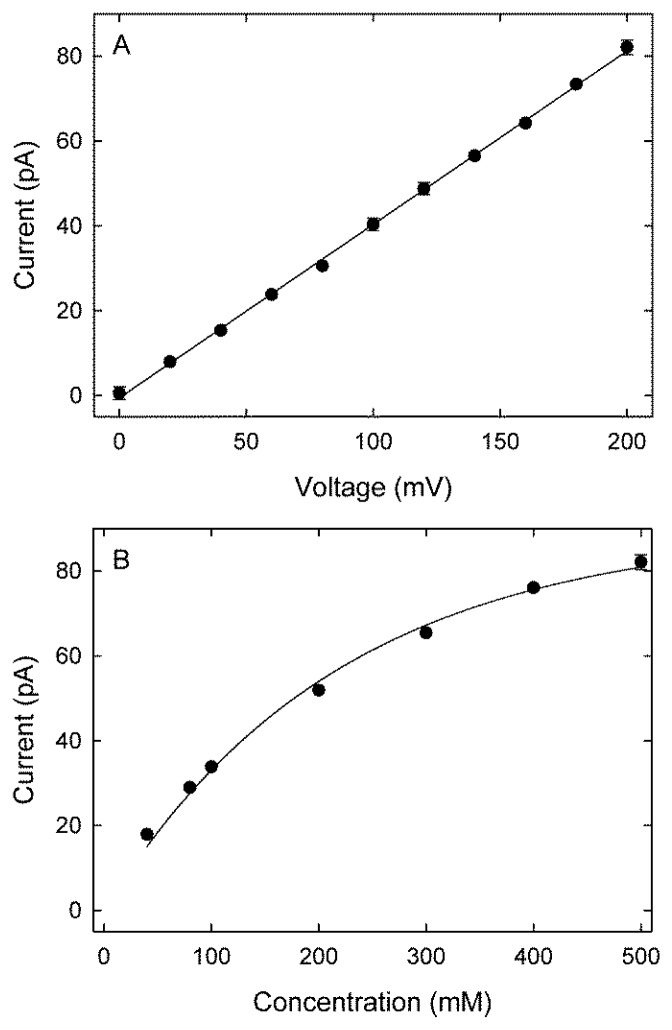
(**A**) Current-voltage profile for potassium ions at an ionic concentration of 500 mM, and (**B**) Current-concentration profile for potassium ions at an applied potential of 200 mV. Data points represent the average of five sets of simulations, each simulation lasting 0.8 μs. Error bars represent one standard error of the mean and error bars smaller than the data points are not shown.

The current-voltage profile for potassium ions for the (9, 9) exohydrogenated CNT, obtained with symmetrical solutions of 500 mM in both reservoirs, is shown in Figure 2(A). The current-voltage relationship is linear, with a current of 82 pA at 200 mV, equivalent to 5.1 × 10^8^ ions per second. No chloride conductance was observed for all investigated voltages and concentrations. The magnitude of current across the nanotube plotted against the ionic concentration of potassium ions in the reservoirs follows the Michaelis-Menten form Figure 2(B). For example, at an applied potential of 200 mV, an ionic concentration of 200 mM reduces the current to 52 pA. The current increases rapidly with an increasing ionic concentration initially and then saturates with a further increase in concentration. The current saturates at approximately 127 pA, and the concentration for the half-maximum current occurs at 277 mM.

A single channel in the ICS™ biosensor [22] has an experimentally measured current of 4 pA at 200 mV [23,24]. The carbon nanotube presented here demonstrates a 20-fold increase in current compared to the ICS™ biosensor. For the nanotube to function as a biosensor it is necessary to block the nanotube pore, preferably when no analyte is present so that we observe conduction when analyte is present. In the next section we investigate two blocker molecules constructed from fluorinated C_60_ fullerenes.

### 3.2. Blockage by Fluorofullerenes

The carbon nanotubes examined in the previous section are permanently open in that monovalent cations move through the pore under the influence of an applied potential. For these nanotubes to form part of a biosensor we want the pore to be closed normally and to open when a specific molecule we wish to detect is present, so that the biosensor is “switched on” upon detection. It is therefore necessary to determine a suitable blocking molecule which can non-covalently bind to the nanotube entrance with sufficient energy that its probability of reopening is low. Moreover, the binding strength must not be too strong such that the blocking molecule cannot be stripped away when the molecule of interest is detected.

We examine two fluorofullerenes, namely C_60_F_36_ and C_60_F_60_, as potential blocking molecules. We assume T symmetry for the C_60_F_36_ fluorofullerene taken from Taylor [46] which is the most stable of the three isomers [47,48]. Fluorinated fullerenes were first synthesized in 1991 by the reaction of molecular fluorine with C_60_ [49]. Fluorofullerene molecules from C_60_F_2_ to C_60_F_102_ have been detected in the gas phase [49]. C_60_F_18_, C_60_F_36_ and C_60_F_48_ are readily produced [38,46] and experimentalists have shown that only species with an even number of fluorine atoms are observed [38]. It is difficult to add more than 48 fluorine atoms to the C_60_ cage, but fullerenes with higher fluorination have been observed in small concentrations such as the C_60_F_60_ fullerene [46,49]. We chose the C_60_F_36_ and C_60_F_60_ fluorofullerenes so as to obtain a range as to the fluorofullerene's potential binding strength to the carbon nanotube. Ab initio calculations have shown that tube-like isomers of C_60_F_60_ are more stable than the cage isomer [50]. In this paper we only study the cage isomer.

**Figure 3.**
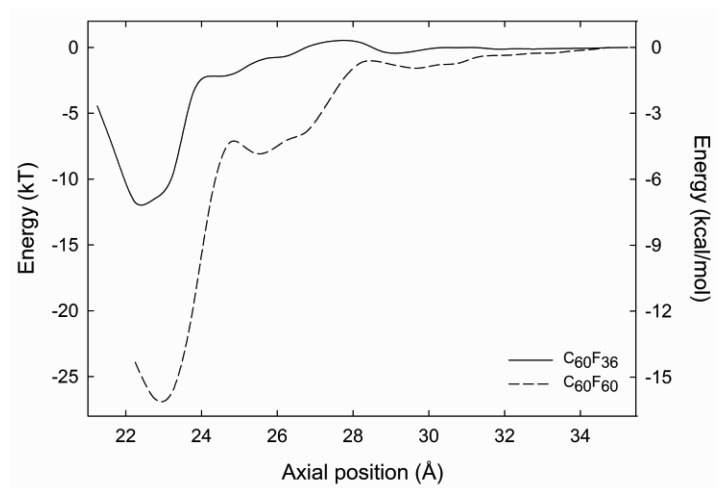
Fluorofullerene PMF. PMF profile for the unbinding of the C_60_F_36_ and C_60_F_60_ fluorofullerenes from the (9, 9) exohydrogenated carbon nanotube.

The centre of mass of the fluorofullerenes are placed 3 Å from the nanotube entrance (22 Å) and after 1 ns equilibrium simulations the fluorofullerenes remain at the nanotube entrance. To verify the predicted binding of these fluorofullerenes to the nanotube we determine the PMF for the unbinding from the nanotube. As shown in Figure 3, the PMF reaches a minimum at 22 Å and 23 Å for the C_60_F_36_ and C_60_F_60_ fullerenes, respectively, where the nanotube centre of mass is at 0 Å. For the C_60_F_60_ fullerene the PMF increases rapidly from −25.6 kT at 23 Å to −7.3 kT at 24.5 Å. The C_60_F_36_ fullerene exhibits a shallower well of −11.9 kT. It is assumed that the properties for the window at 35 Å are similar to bulk, and therefore the PMF is set to zero at this point. Unlike the C_60_F_36_ fullerene, the C_60_F_60_ fullerene also exhibits another shallower minimum energy position at approximately 25.5 Å. At this position the C_60_F_60_ fullerene binds to the edge of the nanotube partially occluding the nanotube entrance. The fluorofullerenes non-covalently bind to the nanotube entrance through favourable electrostatic and van der Waals interactions.

**Figure 4.**
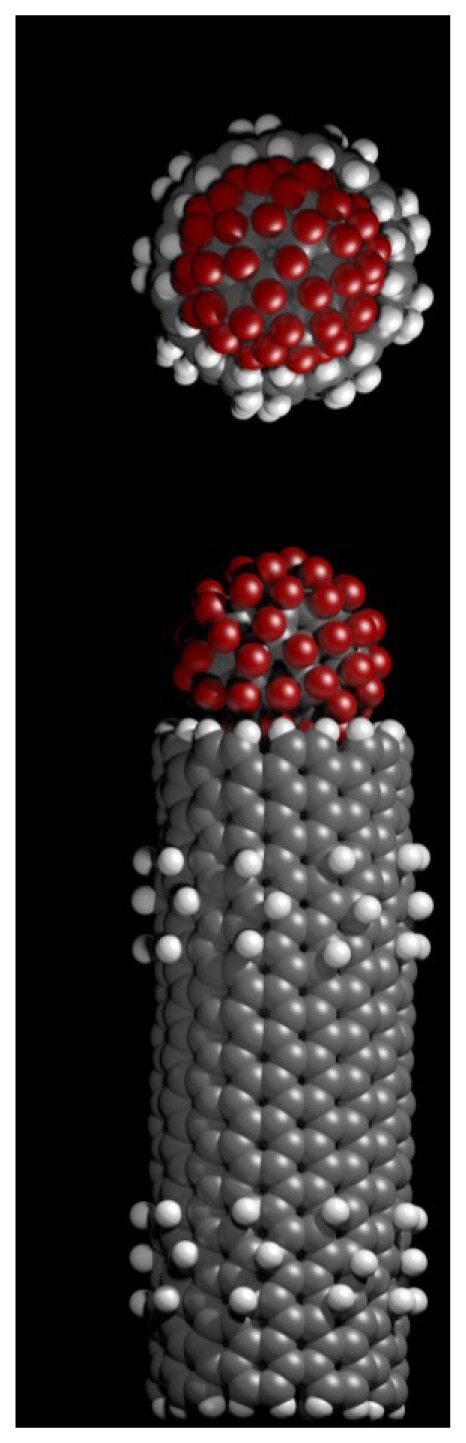
Illustration of the bound configuration of C_60_F_60_ to the carbon nanotube. (**A**) top view and (**B**) side view.

The bound configuration of C_60_F_36_ and C_60_F_60_ demonstrate complete blockage of the nanotube pore. This is illustrated in Figure 4 for the C_60_F_60_ fullerene, a similar blockage is observed for the C_60_F_36_ fullerene. Using Equation (1) we obtain a dissociation constant, K_d_ of 87 μM and 37 pM for the C_60_F_36_ and C_60_F_60_ fullerenes, respectively.

### 3.3. Biosensor Construction and Function

There are three main components of our biosensor design: the carbon nanotube pores embedded in a membrane, a fluorofullerene with a Fab fragment attached to its surface, and a soluble polymer bead with complementary Fab fragments. The function of our biosensor relies on the removal of the fluorofullerene from the nanotube entrance in response to analyte. However, to reduce noise of the system it is important that we choose a fluorofullerene which exhibits a large binding energy such that the probability of channels spontaneously opening and closing is as small as possible. In this section we will describe the construction of our three components and how they fit together to detect analyte.

It is possible to embed the carbon nanotubes into a highly dense array enabling the biosensor the greatest chance of detecting the analyte. For example, our exohydrogenated (9, 9) carbon nanotubes could be embedded in a silicon-nitride membrane similar to the work of Holt *et al.* [51]. They obtained a pore density of 2.5 × 10^11^ cm^−2^ in their carbon nanotube-silicon nitride membrane. However, their membrane thickness is 2–3 μm. To make our theoretical calculations tractable computationally we use a short tube. For the nanotubes we examine in this paper to span this thickness we must increase their length by approximately 1,000 times. But a longer nanotube will essentially produce the same results once we exohydrogenate sections along its entire length. For instance, the PMF for potassium ions would then be an undulating profile along the entire length with multiple energy wells and barriers and a K^+^ would only ever face a 2.2 kT barrier as in Figure 1(A).

From our MD simulations the C_60_F_60_ fullerene exhibits the largest binding energy, with a depth of 25.6 kT (Figure 3). The probability of the nanotube pore opening and closing using this fluorofullerene will be very small, approximately 10^−8^, so that noise in the system will be minimal and we can simply calculate our baseline current and the mean duration a pore stays open once it opens spontaneously. Next, we must attach Fab fragment to the fluorofullerene. Taylor *et al.* [52] demonstrated the possibility of including oxygen atoms on prefabricated fluorofullerenes, where epoxides on the nanotube surface are produced by nucleophilic substitution of F by OH. Moreover, fluorofullerenols, fluorofullerenes with hydroxides attached, are readily formed from C_60_F_36_ by reaction with moist solvent and can be obtained in significant yield [47]. Using this methodology we propose to attach 2–3 hydroxyl groups on the C_60_F_60_ fullerene, such that it will become C_60_F_58_(OH)_2_. These hydroxyl groups can then be used to functionally attach Fab fragments. First, C_60_-PEG conjugation will be performed on the fluorofullerenols as previously reported [53,54] with biotin attached to the PEG chain. This biotin-PEG chain would then be coupled to a biotinylated antibody fragment (Fab) using streptavidin intermediates, similar to that described by Cornell *et al.* [22]. These fluorofullerene conjugates would be placed on one side of the carbon nanotube membrane.

We propose to utilize polymer beads, with radii of approximately 15 nm [55], which have been widely investigated for use in medicine and biology through to opto-electronics [55]. The surface of the soluble polymer beads will be functionalized with antibodies or complementary Fab fragments to that on the fluorofullerene with synthesis according to the procedures described by Uchida *et al.* [56], van Erp *et al.* [57], Sarobe *et al.* [58], and Kawaguchi [55]. For example, Sarobe *et al.* [58] covalently coupled immunoglobulin G (IgG) to chloromethylstyrene beads to create stable sensitized latex beads. Uchida *et al.* [56] immobilized both intact IgG or Fab fragments of antibovine serum albumin antibody to polymer microspheres using an oxidized dextran as a binder. Moreover, DNA has been immobilized on polymer beads using biotin-avidin binding [55]. The beads will be placed on the same side of the membrane as the fluorofullerene conjugates.

**Figure 5.**
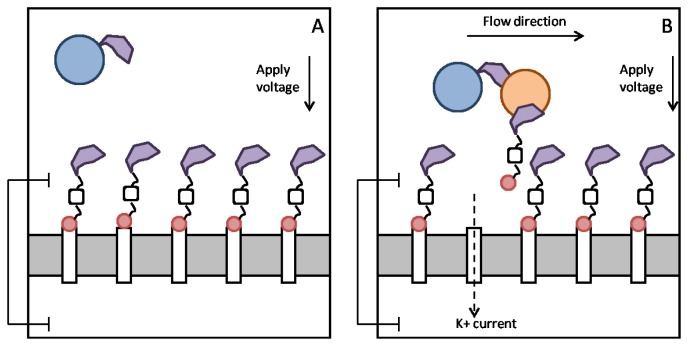
Schematic representation of the carbon nanotube-fluorofullerene biosensor. (**A**) In the absence of analyte (orange) all nanotube pores are blocked by fluorofullerene (red)—Fab fragment (purple) conjugates. (**B**) Analyte is injected into the sample, and binds to the Fab fragments on the fluorofullerene and polymer bead (blue) floating in solution. The flow from the injected analyte solution is sufficient to pull a fluorofullerene from its docked position and potassium current is observed.

When no analyte is present in the top reservoir, the fluorofullerene conjugates will block the entrances to the nanotube pores and the polymer beads will float around Figure 5(A). When analyte is injected into the top reservoir the Fab fragments present on the polymer bead and fullerene conjugate will crosslink to the analyte. The flow caused by the injection of analyte will act to pull the fluorofullerene from the nanotube entrance due to the drag forces on the polymer bead. This will in turn unblock the pore, as illustrated in Figure 5(B), and a potassium current will be detected and thus the analyte. Using the present design it would not be possible to detect multiple analytes with distinct binding affinities, such as a low or high affinity analyte. The current flowing across the open nanotubes may be monitored with a conventional current-to-voltage amplifier, such as the one used routinely for measuring ionic channel currents. If finer resolutions are required, we can make use of the signal processing techniques based on the hidden Markov model [59] or fluctuation analyses [60,61]. To open a pore, the drag force *F_D_* on these polymer beads must be sufficient to pull the fluorofullerenes from the nanotube entrance. In other words, the drag force must be greater than the fluorofullerene-nanotube binding energy. If we assume laminar flow past a sphere, Stokes Law states that *F_D_* = *6πμaU*, where *a* is the radius of the sphere (15 nm), *U* is the flow speed and μ is the viscosity. If we assume an injection speed [62] of 1.2 mL/min and viscosity of water we obtain a drag force of approximately 1.2 × 10^−13^ N for a polymer bead 15 nm in radius. This is many orders of magnitude larger than the binding force of the fluorofullerene which for the C_60_F_60_ fullerene is approximately 3.5 × 10^−20^ N. The binding affinity between the Fab fragments and the analyte must also be equal to or greater than the fluorofullerene-nanotube binding affinity to ensure that the fluorofullerenes can be pulled from the nanotube mouth. We determine dissociation constants of 87 μM and 37 pM for the C_60_F_36_ and C_60_F_60_ fullerenes, respectively. Friguet *et al.* [63] determined the dissociation constant of antigen-antibody equilibria in solution in the nM range. Therefore, to ensure the fluorofullerene can be pulled from the nanotube entrance the fullerene must be functionalized with somewhere between 36 and 60 fluorine atoms. Fullerenes can be fabricated with between 2 and 60 fluorine atoms [47] but the C_60_F_48_ fullerene, which is formed readily [46], may provide the best blocking characteristics.

As described in Section 3.1, a single nanotube channel facilitates the flow of 5.1 × 10^8^ ions per second. This magnitude is significantly larger than through the Ion Channel Switch ICS™ biosensor where a single channel facilitates 2.5 × 10^7^ ions per second [22]. Moreover, assuming a similar pore density to that observed by Holt *et al.* [51], our membrane would exhibit a high channel density. For example, assuming all channels are initially blocked by fluorofullerene conjugates, there would be 2.5 × 10^11^ available recognition sites in a device 1 cm^2^. In work to miniaturize the ICS™ biosensor to 1 μm radius, noise becomes a significant issue since at this size there are only a few ion channels and measured current is of the order of picoamperes [26]. Using the biosensor design in this paper a 1 μm^2^ membrane would contain 2,500 nanotube channels and the measured current per nanotube is an order of magnitude larger. Therefore, although the background noise of our proposed biosensor is not known, it is likely that the sensitivity of our biosensor will be an order of magnitude smaller than the ICS™ biosensor. Furthermore, at voltages above 150–200 mV the ICS™ biosensor will not function since the membrane will break down. In contrast, voltages as high as 1 V could be applied to the fluorofullerene-nanotube biosensor further increasing the current flow through the tube. Some of the channels in the membrane of the ICS™ biosensor can diffuse laterally within the plane of the membrane [22]. The antibodies on the mobile channels scan an area of 1 μm^2^ in less than 5 min, and the speed and sensitivity of the biosensor response is proportional to the number of binding sites accessible to each mobile channel [22]. In other words, the sensitivity to analyte is enhanced since each gramicidin channel is able to cross link with any of many sites at the sensor surface to which the molecule to be detected may bind. In contrast, the fluorofullerene-carbon nanotube biosensor proposed here would be constructed of nanotubes fixed into their membrane, thereby removing the sensitivity gained from in-plane diffusion. However, as mentioned our membrane would exhibit a high density of channels which would improve its sensitivity due to the large number of recognition sites.

## 4. Conclusions

Using both molecular and stochastic dynamics, we show that an exohydrogenated (9, 9) carbon nanotube with hydrogen terminated ends exclusively conducts monovalent cations with a current of 82 pA at 200 mV. In comparison gramicidin-A conducts potassium ions with an experimentally measured current of 4 pA at 200mV [23,24]. Furthermore, we demonstrate that it is possible to completely block the nanotube pore using fluorofullerenes, with dissociation constants of 87 μM and 37 pM for the C_60_F_36_ and C_60_F_60_ fullerenes, respectively.

We propose at the proof-of-concept level the design of a biosensor comprised of the functionalized carbon nanotube pores embedded in a membrane, a fluorofullerene with a Fab fragment attached to its surface, and a soluble polymer bead with complementary Fab fragments. Under normal operating conditions, when no analyte is present, the nanotubes are blocked by a C_60_F_60_ fluorofullerene-Fab conjugate and the polymer beads float around in the reservoir. When analyte is injected into the top reservoir the Fab fragments present on the polymer bead and fullerene conjugate crosslink to the analyte. Then, the drag force on the much larger polymer bead will act to pull the fluorofullerene away from the nanotube and unblock the pore and thus allow the flow of monovalent cations across the membrane.

The advantages of this carbon nanotube fluorofullerene biosensor compared to the ICS™ biosensor are: (i) a 20-fold increase in potassium ion conductance, (ii) the potential to activate a large number of channels since the nanotubes can be densely packed into membrane, and (iii) the potential to develop a more sensitive current amplifier since current can be measured directly. Furthermore, assuming a tight seal is formed between the two reservoirs such a biosensor would be able to detect the flow of currents caused by the opening of only a few nanotubes and thus a few molecules of analyte making it a highly sensitive detection design.

Much work needs to be done before such a device is realized. For example, as a proof-of-concept we have examined only one nanotube diameter and terminal functionalization. Future work will be necessary to study the effect of nanotube diameter, and carbon nanotube tip functionalization on the fullerene binding energy. It is now possible to fabricate hydrogenated carbon nanotubes using an atomic hydrogen beam, and these functionalized nanotubes have been shown to be stable at room temperature [64]. Unfortunately, lack of control of functionalization location remains a challenge. However, Raghuveer *et al.* [65] demonstrated the ability to site-selectively functionalize the outside surface of multi-walled carbon nanotubes using focused-ion-beam irradiation and subsequent mild chemical treatments. Further work is needed in this area to facilitate the creation of devices such as our proposed biosensor.
